# Real‐World Nonmotor Changes in Patients with Parkinson's Disease and Motor Fluctuations: J‐FIRST

**DOI:** 10.1002/mdc3.12939

**Published:** 2020-04-11

**Authors:** Hirohisa Watanabe, Hidemoto Saiki, Shih‐Wei Chiu, Takuhiro Yamaguchi, Kenichi Kashihara, Yoshio Tsuboi, Masahiro Nomoto, Nobutaka Hattori, Tetsuya Maeda, Yasushi Shimo, the J‐FIRST Group

**Affiliations:** ^1^ Department of Neurology Fujita Health University Aichi Japan; ^2^ Department of Neurology, Kitano Hospital The Tazuke Kofukai Medical Research Institute Osaka Japan; ^3^ Division of Biostatistics Tohoku University Graduate School of Medicine Miyagi Japan; ^4^ Department of Neurology Okayama Kyokuto Hospital Okayama Japan; ^5^ Department of Neurology, Faculty of Medicine Fukuoka University Fukuoka Japan; ^6^ Department of Neurology and Clinical Pharmacology Ehime University Graduate School of Medicine Ehime Japan; ^7^ Department of Neurology Juntendo University School of Medicine Tokyo Japan; ^8^ Division of Neurology and Gerontology, Department of Internal Medicine, School of Medicine Iwate Medical University Iwate Japan; ^9^ Department of Neurology and Movement Disorder Research Research Institute for Brain and Blood Vessels‐Akita Akita Japan

**Keywords:** Parkinson's disease, nonmotor symptoms, Movement Disorder Society Unified Parkinson's Disease Rating Scale (MDS‐UPDRS), observational study, Japan

## Abstract

**Background:**

Nonmotor symptoms (NMSs) of Parkinson's disease (PD) impair health‐related quality of life.

**Objectives:**

To identify changes in NMSs during 52 weeks in Japanese PD patients exhibiting motor fluctuations.

**Methods:**

In PD patients with ≥1 NMS and wearing‐off, changes in total/subscore of the Movement Disorder Society Unified PD Rating Scale (MDS‐UPDRS) Part I and 8‐item PD Questionnaire were assessed. Group‐based trajectory models were used to characterize longitudinal patterns of MDS‐UPDRS Part I.

**Results:**

Data from 996 patients were analyzed. MDS‐UPDRS Part I subscores for cognitive function decreased linearly over time. Total and subscores for apathy and lightheadedness on standing significantly deteriorated with fluctuations, whereas other subscores fluctuated without significant deterioration. Changes in the MDS‐UPDRS Part I total score correlated with changes in the 8‐item PD Questionnaire total score. Based on group‐based trajectory models, longitudinal pattern analysis of MDS‐UPDRS Part I scores yielded the following 3 separate groups: unchanged (63.8%), deteriorated (20.1%), and improved (16.2%). The improved group had significantly more NMSs at baseline, significantly higher MDS‐UPDRS Part I/8‐item PD Questionnaire total scores, and modified Hoehn and Yahr scores, and had received treatment for NMSs. The multivariate analysis revealed significant associations between severe motor disability and receiving any treatment for NMSs at baseline and improvement of MDS‐UPDRS Part I total scores.

**Conclusions:**

Changes in MDS‐UPDRS Part I scores were variable and related to changes in health‐related quality of life in PD patients with motor fluctuations.

Nonmotor symptoms (NMSs), including pain and dysautonomia, as well as cognitive, neuropsychiatric, and sensory symptoms are an integral aspect of the clinical presentation of Parkinson's disease (PD).^1^ Generally, NMSs are observed in both the prodromal and advanced phases of PD. Compared with motor symptoms, NMSs have a greater negative impact on the health‐related quality of life (HrQOL) of PD patients, yet the clinical relevance of NMSs may be underestimated in clinical practice.^2^ The Parkinson and Non Motor Symptoms (PRIAMO) study demonstrated that overall NMS progression does not follow motor deterioration, is symptom specific, and only the development of specific domains negatively impacts quality of life.^3^

In the clinical setting, advanced PD patients with motor fluctuations tend to present with NMSs more frequently than nonadvanced patients, and the NMSs themselves tend to be more severe. The recognition, quantification, and appropriate management of NMSs have become relevant concerns in clinical practice, particularly in advanced PD. Furthermore, NMSs are considered the basis for the future development of disease‐modifying therapy for prodromal PD.^4^

Risk factors for NMSs and NMS‐dominant subtypes of PD are heterogeneous.^5^ Deficiencies of dopamine and other neurotransmitters in the central and peripheral nervous systems are reportedly involved in the development of NMSs PD.^6^ Furthermore, the “wearing‐off” motor fluctuations that occur with long‐term levodopa treatment seem to occur simultaneously with NMSs.^7^ Thus, interactions between NMSs and the adverse effects of currently available PD treatments are also relevant.^8^ A real‐world prospective assessment of the natural history of NMSs and their association with HrQOL is necessary to achieve better management of NMSs. Particularly, little evidence is available on the patterns of change in NMSs over time.

J‐FIRST is a large‐scale observational study evaluating NMSs in PD patients with motor complications and the relationship between NMSs and HrQOL; the baseline data of which have been previously published.^9^ This study aimed to identify the changes in NMSs over time in a real‐world clinical setting using the J‐FIRST data set. In addition, we assessed the relationship between changes in NMSs and HrQOL and sought to identify the associated clinical factors.

## Methods

### Study Design and Setting

This prospective observational study was conducted in 35 sites (Supplementary Text S1) throughout Japan between February 2014 and December 2016. PD patients who met the eligibility criteria were enrolled between March 2014 and January 2015 at participating medical centers and were prospectively investigated for 52 weeks.

### Eligibility Criteria

The detailed diagnostic and eligibility criteria were previously reported.^9^ Briefly, we enrolled PD patients presenting wearing‐off motor fluctuations with levodopa‐containing drugs and ≥ 1 NMS assessed by the Movement Disorder Society Unified Parkinson's Disease Rating Scale (MDS‐UPDRS) Part I.

### Endpoints

The primary endpoints were the interval changes over 52 weeks in the MDS‐UPDRS Part I^10^ and 8‐item PD Questionnaire (PDQ‐8),^11^ both of which were validated and translated into Japanese. The Modified Hoehn and Yahr (mH&Y) scale was used to measure overall motor disability in the *on* and *off* states.

### Assessments

Patient clinical characteristics data were obtained by a structured interview and neurological examination at baseline. The presence and severity of NMSs were evaluated using the MDS‐UPDRS Part I. Motor complications were evaluated using the MDS‐UPDRS Part IV. To assess overall motor disability, the patients described their worst state to the attending physician (a neurologist experienced in treatment of movement disorders). The physician judged the level of mH&Y score based on the patients’ descriptions. The PDQ‐8 was used to evaluate HrQOL. Patients continued to undergo evaluations at every subsequent visit during the 52‐week study period (6 visits in total).

Treatments for PD and NMSs were recorded, including daily doses of levodopa‐containing drugs, dopamine agonists, dopamine economizers, and nondopaminergic agents as well as methods of functional neurosurgery (if applicable). Physicians prescribed treatment to ameliorate NMSs at their own discretion. In this study, we analyzed the available data based on whether patients were receiving medications for NMSs at the start of the observation period.

### Statistical Analysis

A target sample size of 1000 PD patients with motor fluctuation was planned based on feasibility and the number of patients required for the identification of the factors related to NMSs. MDS‐UPDRS Part I and its subscores were validated for NMSs in 94 PD patients.^12^ A more recent study including 423 de novo PD patients showed statistically significant changes of MDS‐UPDRS Part I total score and its subscores at 1‐year and 2‐year follow‐up periods.^13^ Although this was an exploratory study, we estimated that 1000 patients would provide sufficient power to detect changes in MDS‐UPDRS Part I and its subscores.

The statistical analysis was independently performed by S.C. and T.Y. using SAS software (version 9.4; SAS Institute Inc., Cary, NC). For all analyses, tests were 2‐sided, and the significance level was 5%. For the exploratory aspects, multiplicity adjustment was not performed. The study started at visit 2 and ended at visit 6. The interval between each visit was 13 weeks. The most recent information collected at each visit was used for the analyses.

The prevalence is shown using frequency and percentage, and the MDS‐UPDRS Part I and PDQ‐8 scores are shown by sample size and mean ± standard deviation. The levodopa‐equivalent dose was calculated using a conversion formula.^14^

Longitudinal changes from baseline in MDS‐UPDRS Part I and its subscores and PDQ‐8 (total score) were estimated by a general linear model using robust variance estimation with compound symmetry covariance structures for repeated measurements. Associations between the MDS‐UPDRS Part I and the PDQ‐8 were evaluated by Pearson's correlation coefficient.

Change patterns of MDS‐UPDRS Part I total scores were clustered into groups using group‐based trajectory models. This method was found to be superior for identifying underlying longitudinal trajectories.^15^ The shape of the pattern was considered by clinical judgment, and the number of groups was chosen by the following rules: using the smaller Bayesian Information Criterion, each group had ≥5% of the total participants and ≥ 90% prediction rate for each group. Although the evidence remains scarce for the treatment of NMSs in PD,^16^ the available data indicate that dopaminergic treatment may influence NMS response.^8^ As the covariate (change of medication) is time‐varying, it is influenced by previous outcome (NMSs); as such, any adjustment for time‐varying covariates after baseline intervention should be made with caution, and this is beyond the capability of conventional regression models.^17^, ^18^ Thus, the present analysis was focused on considering the baseline covariates’ influence rather than the longitudinal changes of covariates to clarify the real‐world situation. Demographic variables between groups were compared by analysis of variance and chi‐squared test. The trend of administration rate of concomitant medications overall and in each group was examined by Cochran–Armitage test. Finally, the relationship between groups and 26 baseline demographic variables (ie, sex, age, smoking history, disease duration of PD, age of onset, *on* state mH&Y, *off* state mH&Y, daily dose of levodopa‐containing drugs, surgical treatment for PD, dyskinesia, caregiver, complications, MDS‐UPDRS Part IV score, and the use of ergot dopamine agonists, other dopamine agonists, pramipexole, rotigotine, ropinirole, entacapone, selegiline, zonisamide, istradefylline, droxidopa, amantadine, anticholinergic agents, or medication for NMSs) was evaluated by a multivariate logistic regression model. Although some of the 26 variables seem very similar, they have different clinical meanings, and the correlation coefficient between them was not very high. Odds were calculated as the number of people in the improved group divided by the number of people in the deteriorated group. The odds ratio expressed the relative chance of being in the “improved group” under a specified level of demographic variable compared with the reference level of the demographic variable.

## Results

### Patient Disposition and Baseline Characteristics

Initially, 1021 patients were registered. Of these, 3 patients did not meet the eligibility criteria, 5 withdrew consent, and 5 were ineligible to continue because of hospitalization, death, or difficulty in evaluation at baseline. Of the 1008 enrolled patients, 12 did not present wearing‐off under treatment with levodopa‐containing drugs, as determined by MDS‐UPDRS Part IV. As a result, 996 patients were included in the final analysis.

Of 996 patients, 624 (62.3%) were women and 372 (37.7%) were men. The mean age was 68.1 years, and the median duration of illness was 10.9 years. The mean MDS‐UPDRS Part I and PDQ‐8 scores were 10.9 and 7.3, respectively, and the mean number of NMSs was 6.6. All patients had been treated with levodopa‐containing drugs. The mean ± standard deviation levodopa‐equivalent dose was 769.5 ± 339.0 mg/day (Table 1). Further details of patient baseline characteristics were described previously.^9^

**Table 1:** *Clinical characteristics at baseline (n = 996)*

Sex, male/female, %	37.7/62.3
Age, y, mean ± SD (age category [%])	68.1 ± 8.78 (60–69 [37.1], 70–79 [40.4])
Duration of PD, y, mean ± SD	10.9 ± 5.5
Onset age of PD, y, mean ± SD (age category [%])	58.2 ± 9.94 (50–59 [34.4], 60–69 [34.4])
Number of nonmotor symptoms, mean ± SD	6.6 ± 2.5
Duration of motor symptoms, y, mean ± SD	8.8 ± 5.3
History of surgical treatment for Parkinson's disease, %	3.5
Dyskinesia, %	45.3
*On* state mH&Y, mean ± SD (%)*	1.6 ± 0.6 mild (45.4), moderate (48.5)
*Off* state mH&Y, mean ± SD (%)*	2.3 ± 0.6 moderate (54.9), severe (35.8)
MDS‐UPDRS Part IV score, mean ± SD	1.4 ± 0.7
MDS‐UPDRS Part I total score, mean ± SD	10.9 ± 5.37
Total score of PDQ‐8, mean ± SD	7.3 ± 5.2
Use of levodopa‐containing drugs, %	100.0
Daily dose of levodopa‐containing drugs (mg), mean ± SD	436.4 ± 165.7
Daily dose of levodopa‐containing drugs (mg) by tertile, mean ± SD	First: 265.8 ± 68.2 Second: 411.5 ± 36.2 Third: 612.4 ± 108.2
Levodopa‐equivalent dose (mg/day), mean ± SD	769.5 ± 339.0

* Assessed as mild, mH&Y scores 0, 1.5, and 2; moderate, mH&Y scores 2.5 and 3; and severe, mH&Y scores 4 and 5.

mH&Y, modified Hoehn and Yahr scale; MDS‐UPDRS, Movement Disorder Society Unified Parkinson's Disease Rating Scale; PD, Parkinson's disease; PDQ‐8, 8‐item Parkinson's Disease Questionnaire; SD, standard deviation.

### Changes in MDS‐UPDRS Part I Total Score and Subscores over 52 Weeks

**Figure 1:**
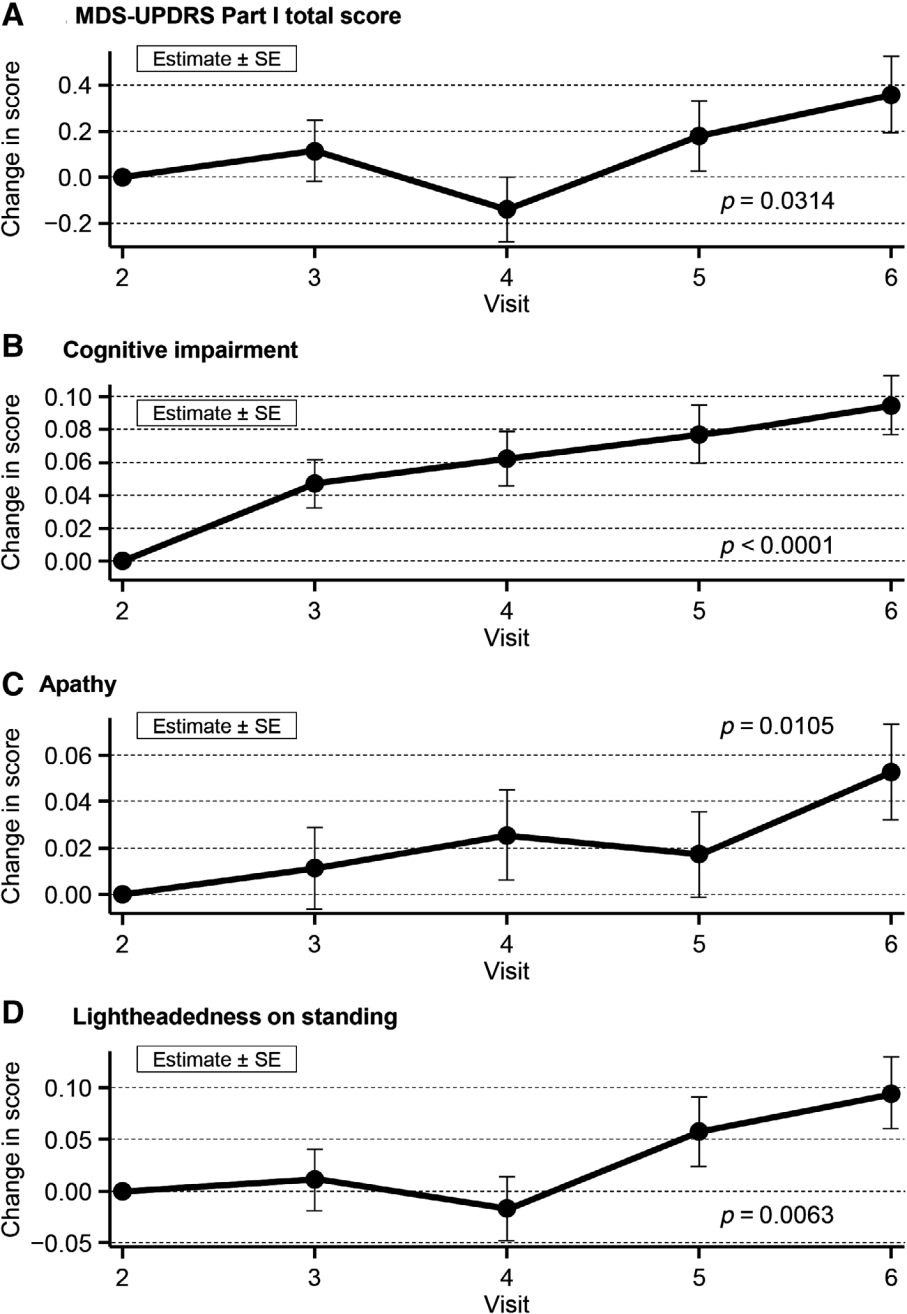
Overall MDS‐UPDRS Part I total score (**A**) and subscores for cognitive impairment (**B**), apathy (**C**), and lightheadedness on standing (**D**). MDS‐UPDRS, Movement Disorder Society Unified Parkinson's Disease Rating Scale; SE, standard error.

The MDS‐UPDRS Part I total score fluctuated over 52 weeks and significantly deteriorated by the end of the study (Fig. 1A). Among all MDS‐UPDRS Part I subscores, cognitive impairment was the only subscore to significantly deteriorate progressively over time (Fig. 1B). Subscores for apathy and lightheadedness on standing significantly deteriorated with fluctuations (Fig. 1C,D), whereas the other subscores fluctuated without significant changes. Compared with baseline, the MDS‐UPDRS Part I total score deteriorated by 0.36 points (*P* = 0.0314), cognitive impairment by 0.09 points (*P* < 0.0001), apathy by 0.05 points (*P* = 0.0105), and lightheadedness on standing by 0.10 points (*P* = 0.0063) at 52 weeks.

In addition, we analyzed the change adjusted for the baseline levodopa equivalent dosage (MDS‐UPDRS Part 1 total score, score of each item, PDQ‐8 total score). Even after adjusting for baseline levodopa equivalent dosage, the change from baseline did not differ much from that before the adjustment, and the effect of the levodopa equivalent dosage was limited. Items with a significant change at week 52 were the same as those with a significant change before the adjustment.

### Changes in PDQ‐8 Score and Correlation with MDS‐UPDRS Part I Total Score

**Figure 2:**
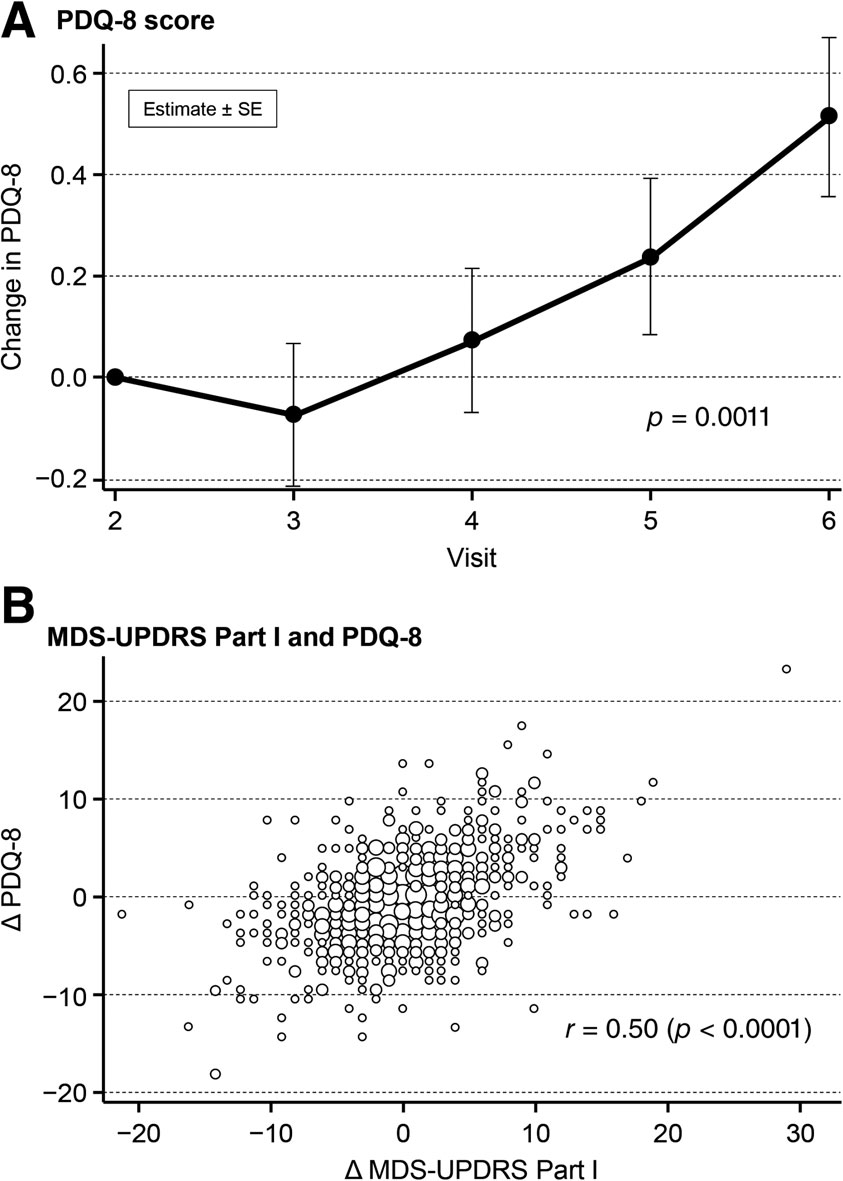
PDQ‐8 score (**A**) and correlation of MDS‐UPDRS Part I total score and PDQ‐8 score (**B**). MDS‐UPDRS, Movement Disorder Society Unified Parkinson's Disease Rating Scale; PDQ‐8, 8‐item Parkinson's Disease Questionnaire; SE, standard error.

At 52 weeks, the PDQ‐8 total score was 0.52 points, significantly higher than the score at baseline (*P* = 0.0011; Fig. 2A). Changes in MDS‐UPDRS Part I scores and PDQ‐8 scores ranged from −22 to 29 and − 19 to 23, respectively. Changes in the MDS‐UPDRS Part I total score significantly correlated with changes in the PDQ‐8 total score (*r* = 0.50, *P* < 0.0001; Fig. 2B). There was a weak correlation between the changes in MDS‐UPDRS Part I and Part IV scores (*r* = 0.22, *P* < 0.0001, Pearson's correlation coefficient).

### Characteristics of Changes in MDS‐UPDRS and Associated Clinical Factors

**Figure 3:**
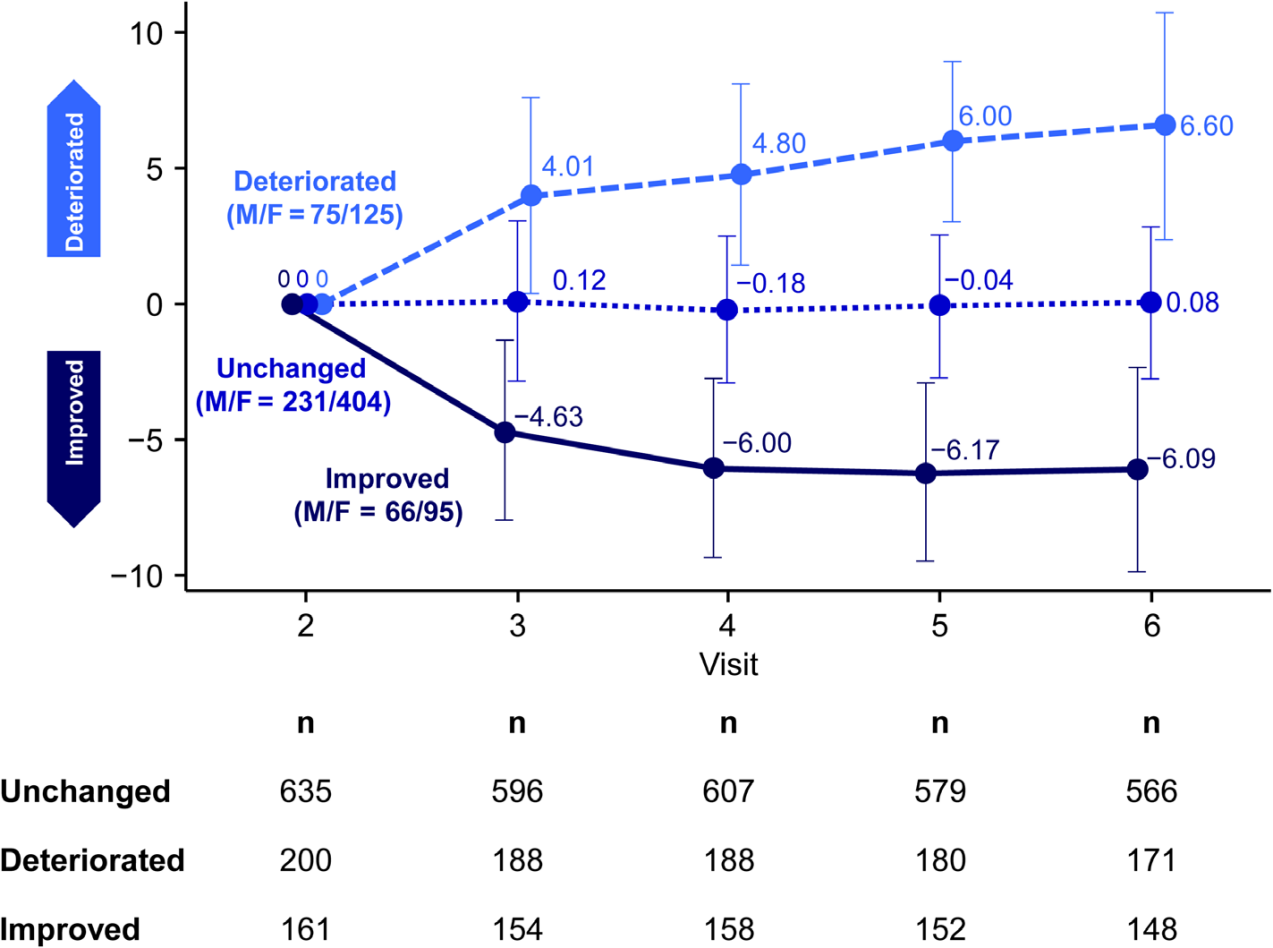
Clustering based on changes in Movement Disorder Society Unified Parkinson's Disease Rating Scale total score. F, female; M, male.

The trajectories of the longitudinal MDS‐UPDRS Part I total scores of each of the 996 patients from baseline to the end of the observation period are shown in Supplementary Fig. S1A; changes in total score are shown in Supplementary Fig. S1B. According to the criteria mentioned in the Methods section, we clustered the change patterns into the following 3 groups: unchanged (n = 635 at visit 2; n = 566 at week 52), deteriorated (n = 200 at visit 2; mean deterioration = 6.60 points and n = 171 at 52 weeks), and improved (n = 161 at visit 2; mean improvement = −6.09 points and n = 148 at week 52; Fig. 3). The number of NMSs (*P* < 0.0001), MDS‐UPDRS Part I total score (*P* < 0.0001), and PDQ‐8 total score (*P* < 0.0001) were significantly higher in the improved group when compared with the unchanged and deteriorated groups. The numbers of NMSs at each time point in the 3 groups (unchanged, deteriorated, and improved) are shown in Supplementary Fig. S2A,B,C, respectively. The proportion of patients with a serious condition based on the mH&Y scale was also significantly higher in both the *on* state (*P* = 0.0004) and the *off* state (*P* = 0.0456).

### Relationship Between Groups and Demographic Variables (Multivariate Logistic Regression)

The multivariate analysis revealed that severe motor disability (*on* state, measured by the mH&Y scale, odds ratio = 4.131, 95% confidence interval = 1.339–12.744; *P* = 0.0136) and any treatment for NMSs at baseline (odds ratio = 1.893, 95% confidence interval = 1.044–3.434; *P* = 0.0356) were significantly associated with improvement of the MDS‐UPDRS Part I total score (Supplementary Fig. S3). In all patients, regardless of the changes in MDS‐UPDRS Part I total score, use of levodopa–carbidopa or levodopa–benserazide decreased over time. However, there was an increase in the use of drug combinations such as levodopa–carbidopa–entacapone and in the use of rotigotine and istradefylline.

## Discussion

In this prospective observational study, we evaluated the longitudinal NMS changes in PD patients with motor fluctuations in a real‐world clinical setting. We observed several novel results. First, changes in MDS‐UPDRS Part I scores were correlated with HrQOL changes in PD patients with motor fluctuations. Second, group‐based trajectory models to characterize longitudinal patterns of the MDS‐UPDRS Part I yielded 3 separate groups: unchanged (n = 635), deteriorated (n = 200), and improved (n = 161). The improved group showed a significantly higher number of NMSs; the MDS‐UPDRS Part I total score, PDQ‐8 total score, and mH&Y scale in both the *on* state and *off* state were significantly higher compared with the unchanged and deteriorated groups. Third, multivariate analysis showed that patients with severe motor disability and those who received treatment for NMSs at the study start had a greater improvement in MDS‐UPDRS Part I total score compared with patients with mild or moderate disability.

Few studies have reported the longitudinal changes of MDS‐UPDRS Part I in PD patients with motor fluctuations. Based on the group‐based trajectory models, we show 3 longitudinal patterns of MDS‐UPDRS Part I scores: unchanged, deteriorated, and improved. With respect to the patients with de novo PD, a previous study showed the association between a longitudinal increase in NMS severity, older age, and lower cerebrospinal fluid Aβ1‐42 at baseline.^13^ Other studies showed that the MDS‐UPDRS Part I score increased by an estimated 0.25 points/year.^19^ However, to our knowledge, no other studies have shown the trajectory curves and spaghetti plots of MDS‐UPDRS Part I. Because PD is heterogeneous, we consider our strategy important because it can show the various courses of the disease.

### Changes in MDS‐UPDRS Part I Scores and HrQOL Changes

We observed a significant relationship between the changes in MDS‐UPDRS Part I scores and those of the PDQ‐8. MDS‐UPDRS Part I evaluates the nonmotor experience of daily living. This scale is significantly correlated with a number of validated scales for NMSs in PD.^12^ According to a large, multicenter, cross‐sectional study of 3206 PD patients, PDQ‐8 scores were significantly related to MDS‐UPDRS Parts I and II, but not to Parts III and IV.^20^ The PDQ‐8 is a prospectively validated and responsive questionnaire for evaluating the HrQOL of patients with PD.^11^ In this study, changes in MDS‐UPDRS Part I scores and PDQ‐8 scores ranged from −22 to 29 and from −19 to 23, respectively. Research by Horváth and colleagues^21^ indicated that the minimal clinically important difference thresholds for the PDQ‐8 summary index were − 5.94 and + 4.91 for detecting improvement and worsening, respectively. For the MDS‐UPDRS Part I, these thresholds were − 2.45 and + 2.64.^22^ We found that each MDS‐UPDRS Part I item showed at least moderate correlations with corresponding individual clinical scales and with composite scores of the corresponding scales. Our results showed that changes in the MDS‐UPDRS Part I total score translate into a significant effect of either enhancement or deterioration of HrQOL in PD. Thus, the MDS‐UPDRS Part I is a promising tool to evaluate NMSs and HrQOL in PD.

### Longitudinal Patterns of MDS‐UPDRS Part I Assessed by Group‐Based Trajectory Models

In this study, we characterized the longitudinal patterns of NMSs in PD patients with motor fluctuation using group‐based trajectory models that were previously reported to be superior for identifying underlying longitudinal trajectories.^15^ The change in the mean value of MDS‐UPDRS Part I from baseline for all participants was not significant over time, but changes in patterns were various and wide between individuals. However, the group‐based trajectory model successfully identified the following 3 groups: unchanged, deteriorated, and improved.

One of our objectives was to distinguish the latent pattern of longitudinal changes in NMSs in real‐life settings. However, we could not rule out the influence of covariates, such as the administration of antiparkinsonism drugs. Thus, we conducted another group‐based trajectory analysis adjusting for 26 baseline covariates (as described in the Methods section). The adjusted model divided the patients into 3 groups as in the original model, and the parameters and proportions of each group were very similar to those of the original groups. These results confirmed that the original model is sufficient to cluster the progress of the NMSs of PD under routine treatment.

### Limitations

As PD patients without motor fluctuation or dementia, or Mini‐Mental State Examination scores <23 were excluded from this study, our results are not applicable to all PD patients. Conversely, it should be clarified whether MDS‐UPDRS Part I is applicable to PD patients with dementia.

The drugs to manage NMSs were prescribed at the discretion of the treating physician, and the types of available drugs and insurance systems, which may influence the treatment strategy for NMSs, will differ from country to country. Thus, the generalizability of the findings is limited. Although further international collaborative studies are needed to address this issue, we believe this study provides the groundwork regarding study design and analysis methods to further investigate the natural history of NMSs in PD.

We only used the MDS‐UPDRS Part I to define the presence of NMSs; however, the MDS‐UPDRS Part I total score has a strong relationship with a composite score of validated scales for the NMSs of PD.

Because this is a real‐world study, medications changed during the observation period as physicians attempted to manage motor fluctuations. Importantly, as patients could be treated over time for each of the NMSs, the natural history for each NMS could be influenced by the treatment of motor symptoms. Because our aim was to elucidate changes in MDS‐UPDRS Part I over time in the real world, we believe that it was more appropriate to use an unadjusted model rather than an adjusted model, which included medication changes. Therefore, the variable course of NMSs found in this study might be, in part, related to medication changes for not only NMS but also for motor symptoms. In addition, NMSs can also influence each other. Thus, other observational studies are required to calculate the power of clinical trials assessing such treatment under conditions of stable medication.

Finally, there was no healthy control group in this study. Age‐matched healthy control data would allow a more detailed understanding of the pathophysiology of the long‐term fluctuations of NMSs in PD.

In conclusion, we found that changes in MDS‐UPDRS Part I scores correlated with HrQOL changes in PD patients with motor fluctuations. Group‐based trajectory models to characterize longitudinal patterns of MDS‐UPDRS Part I scores yielded the following 3 separate groups: unchanged, deteriorated, and improved. Patients with severe motor disability showed greater improvements in the MDS‐UPDRS Part I total score compared with patients with mild or moderate disability.

## Author Roles

(1) Research Project: A. Conception, B. Organization, C. Execution; (2) Statistical Analysis: A. Design, B. Execution, C. Review and Critique; (3) Manuscript Preparation: A. Writing of the First Draft, B. Review and Critique. C. Final Approval

H.W.: 1C, 3B, 3C

H.S.: 1C, 3B, 3C

S.C.: 2A, 2B, 2C, 3B, 3C

T.Y.: 2A, 2B, 2C, 3B, 3C

K.K.: 1C, 3B, 3C

Y.T.: 1C, 3B, 3C

M.N.: 1A, 1B, 1C, 3B, 3C

N.H.: 1A, 1B, 1C, 3B, 3C

T.M.: 2C, 3A, 3B, 3C

Y.S.: 1C, 3B, 3C

## Disclosures

**Ethical Compliance Statement:** Study approval was granted by the Ethics Review Committee at each study site. All patients provided written informed consent prior to participation. We confirm that we have read the Journal's position on issues involved in ethical publication and affirm that this work is consistent with those guidelines. The study was registered in http://clinicaltrials.gov (NCT02073981) and the University Hospital Medical Information Network (UMIN) Clinical Trials Registry (http://umin.ac.jp/ctr/index‐j.htm; UMIN000013161).

**Funding Sources and Conflict of Interest:** This study was sponsored by Kyowa Kirin Co. Ltd., Editorial support was provided by Keyra Martinez Dunn, MD, of Edanz Medical Writing on behalf of EMC K.K., which was funded by Kyowa Kirin Co. Ltd. H.W., K.K., and T.M. received lecture fees from Kyowa Kirin Co. Ltd. H.S. and S.C. received lecture fees and honoraria from Kyowa Kirin Co. Ltd. T.Y. received research support from Kyowa Kirin Co. Ltd. Y.T. received lecture fees and research support from Kyowa Kirin Co. Ltd. M.N. received honoraria from Kyowa Kirin Co. Ltd. N.H. received consulting fees, lecture fees, honoraria, and grants from Kyowa Kirin Co. Ltd. Y.S. received lecture fees, research support, and grants from Kyowa Kirin Co. Ltd.

**Financial Disclosures for Previous 12 Months:** H.W. received lecture fees from Otsuka Pharmaceutical, Kyowa Kirin, Takeda Pharmaceutical, Sumitomo Dainippon Pharma, FP Pharmaceutical, Eisai, Daiichi Sankyo, and Novartis Pharma, and grants from Otsuka Pharmaceutical and FUJIFILM RI Pharma H.S. received lecture fees from Sumitomo Dainippon Pharma, Otsuka Pharmaceutical, Kyowa Kirin, Medtronic Inc., Takeda Pharmaceutical, Nippon Boehringer Ingelheim, Novartis Pharma, M3 Inc., Eisai, GlaxoSmithKline, and Nihon Medi‐Physics; honoraria from Elsevier Japan and Japan Medical Journal; research support from Kyowa Kirin; and grants from Otsuka Pharmaceutical and Nippon Boehringer Ingelheim S.C. received lecture fees and honoraria from Kyowa Kirin T.Y. owns stock in Statcom, received lecture fees from Kowa, Nippon Zoki Pharmaceutical, Japan Tobacco, Taiho Pharmaceutical, and EPS Corporation; honoraria from Ono Pharmaceutical; research support from Kyowa Kirin; and grants from EPS Corporation, Nifix, Kowa, MSD, Pharma Consulting Group, Statcom, Sumitomo Dainippon Pharma, Ono Pharmaceutical, AC Medical, A2 Healthcare, FMD K&L Japan, CAC Croit, Japan Tobacco, Japan Media Corporation, Medidata Solutions, and Luminary Medical. K.K. received lecture fees from Boehringer Ingelheim, Kyowa Kirin, Novartis Pharma, Otsuka Pharmaceutical, Sumitomo Dainippon Pharma, FP Pharmaceutical, Nihon Medi‐Physics, and Eisai; and honoraria from Sumitomo Dainippon Pharma. Y.T. received lecture fees from Japan Blood Products Organization, Daiichi Sankyo, Mitsubishi Tanabe Pharma, Takeda Pharmaceutical, Meiji Seika Pharma, Ono Pharmaceutical, Nihon Medi‐Physics, Dainippon Sumitomo Dainippon Pharma, Novartis Pharma, Otsuka Pharmaceutical, and Kyowa Kirin; and research support from Otsuka Pharmaceutical, Kaketsuken, Eisai, Nippon Boehringer Ingelheim, Daiichi Sankyo, Japan Blood Products Organization, Kyowa Kirin, Sumitomo Dainippon Pharma, and Tsumura & Co. M.N. received consulting fees from Hisamitsu Pharmaceutical and Sumitomo Dainippon Pharma; grants from the Japanese Ministry of Health, Labour and Welfare; and honoraria from Sumitomo Dainippon Pharma, Otsuka Pharmaceutical, Kyowa Kirin, Novartis Pharma, and Ono Pharmaceutical. N.H. received consulting fees from GlaxoSmithKline, AbbVie, Eisai, Otsuka Pharmaceutical, Sumitomo Dainippon Pharma, Kyowa Kirin, Hisamitsu Pharmaceutical, Meiji Seika Pharma, Ono Pharmaceutical and FP Pharmaceutical; lecture fees from MSD, Eli Lilly Japan, Eisai, FP Pharmaceutical, Otsuka Pharmaceutical, Tsumura & Co., Kyowa Kirin, GlaxoSmithKline, Takeda Pharmaceutical, Mitsubishi Tanabe Pharma, Nihon Medi‐Physics, Novartis Pharma, Pfizer Japan, Nippon Boehringer Ingelheim, Sumitomo Dainippon Pharma, and Daiichi Sankyo; honoraria from FP Pharmaceutical, Novartis Pharma, Kyowa Kirin, and AbbVie; research support from Otsuka Pharmaceutical; and grants from Astellas Pharma, Eisai, GlaxoSmithKline, Dainippon Sumitomo Pharma, Takeda Pharmaceutical, Novartis Pharma, Pfizer Japan, Kyowa Kirin, Medtronic, Nippon Boehringer Ingelheim, Boston Scientific, Kissei Pharmaceutical and Otsuka Pharmaceutical. T.M. received lecture fees from Kyowa Kirin, Otsuka Pharmaceutical, Sumitomo Dainippon Pharma, and Novartis Pharma. Y.S. received lecture fees from Medtronic, Sumitomo Dainippon Pharma, Kyowa Kirin, Otsuka Pharmaceutical, Novartis Pharma, Boston Scientific, and Nihon Medi‐Physics; research support from Kyowa Kirin; and grants from Medtronic, Kyowa Kirin, Nippon Boehringer Ingelheim, Boston Scientific Corporation, and Kissei Pharmaceutical.

## Supporting information


**FIG. S1** Trajectories of individual MDS‐UPDRS Part I total scores (**A**) and changes in total scores (**B**) from baseline to the end of the observation period in all patients (n = 996). MDS‐UPDRS, Movement Disorder Society Unified Parkinson's Disease Rating Scale.
**FIG. S2**. Number of NMSs at each time point by the following 3 groups: unchanged (**A**), deteriorated (**B**), and improved (**C**). NMSs, nonmotor symptoms.
**FIG. S3**. Odds ratio with 95% Wald confidence limits (multivariate analysis). LD, levodopa‐containing drug; MDS‐UPDRS, Movement Disorder Society Unified Parkinson's Disease Rating Scale; mH&Y, modified Hoehn and Yahr scale; PD, Parkinson's disease.

## Appendix A.

**J‐FIRST Study Group Investigators:** Seiji Kikuchi, MD, PhD (National Hospital Organization Hokkaido Medical Center, site investigator); Kazunori Itoh, MD, PhD (Iwamizawa Neurology Clinic, site investigator); Masahiko Tomiyama, MD, PhD (Aomori Prefectural Central Hospital, site investigator); Takashi Abe, MD, PhD (Abe Neurology Clinic, site investigator); Atsushi Takeda, MD, PhD (National Hospital Organization Sendai‐Nishitaga Hospital, site investigator); Tetsuya Maeda, MD, PhD (Iwate Medical University, Research Institute for Brain and Blood Vessels‐Akita, Site Investigator, trial/publication steering committee member); Takuhiro Yamaguchi, PhD (Tohoku University Graduate School of Medicine, statistician, trial/publication committee member); Shih‐Wei Chiu, MS (Tohoku University Graduate School of Medicine, Statistician, trial/publication committee member); Koichi Hirata, MD, PhD (Dokkyo Medical University, site investigator); Hideo Mori, MD, PhD (Juntendo University Koshigaya Hospital, site investigator); Shigeki Hirano, MD, PhD (Chiba University Graduate School of Medicine, site investigator); Satoshi Kamei, MD, PhD (Nihon University School of Medicine, site investigator); Mutsumi Iijima, MD, PhD (Tokyo Women's Medical University School of Medicine, site investigator); Hiroshi Nagayama, MD, PhD (Nippon Medical School Graduate School of Medicine, site investigator); Nobutaka Hattori, MD, PhD (Juntendo University Hospital, principal investigator, trial/publication committee member); Yasushi Shimo, MD, PhD (Juntendo University Hospital, site investigator, trial/publication committee member); Miho Murata, MD, PhD (National Center of Neurology and Psychiatry, site investigator); Hideto Miwa, MD, PhD (Juntendo University Nerima Hospital, site investigator); Kazuko Hasegawa, MD, PhD (National Hospital Organization Sagamihara National Hospital, site investigator); Kazutoshi Nishiyama, MD, PhD (Kitasato University School of Medicine, site investigator); Hirohisa Watanabe, MD, PhD (Nagoya University, principal investigator, trial/publication steering committee member); Mizuki Ito, MD, PhD (Nagoya University Graduate School of Medicine, site investigator); Tatsuya Hattori, MD, PhD (Honmachi Clinic, site investigator); Ryosuke Takahashi, MD, PhD (Kyoto University Graduate School of Medicine site investigator); Yoshihisa Tatsuoka, MD, PhD (Tatsuoka Neurology Clinic, site investigator); Hidemoto Saiki, MD, PhD (Kitano Hospital, site investigator, trial/publication steering committee member); Takanori Hazama, MD, PhD (Osaka General Medical Center, site investigator); Makio Takahashi, MD, PhD (Osaka Red Cross Hospital, site investigator); Masahito Mihara, MD, PhD (Osaka University Graduate School of Medicine, site investigator); Hirofumi Kusaka (Kansai Medical University, site investigator); Kenichi Kashihara, MD, PhD (Okayama Kyokuto Hospital, site investigator, trial/publication steering committee member); Junji Yoshinaga, MD, PhD (Yoshinaga Neurology Clinic, site investigator); Takafumi Miyachi, MD, PhD (Yanai Medical Center, site investigator); Mitsutoshi Yamamoto, MD, PhD (Takamatsu Neurology Clinic, site investigator); Masahiro Nomoto, MD, PhD (Ehime University Hospital, trial steering committee chair, publication steering committee member); Yoshio Tsuboi, MD, PhD (Fukuoka University Hospital, site investigator, trial/publication steering committee member); Ryoichi Kurisaki, MD (National Hospital Organization Kumamoto‐Minami National Hospital, site investigator); Ryuichi Ohkubo, MD, PhD (Fujimoto General Hospital, site investigator); Ryuji Saigo, MD (Fujimoto General Hospital, site investigator); Kazuo Toda, MD, PhD (Toda Internal Medicine & Rehabilitation Clinic, site investigator).
