# Supplementary material for: Real‐World Nonmotor Changes in Patients with Parkinson's Disease and Motor Fluctuations: J‐FIRST
Source: Mov Disord Clin Pract. 2020 Apr 11;7(4):431–9. doi: 10.1002/mdc3.12939 (PMC7197319; doi:10.1002/mdc3.12939)
Supplement: Supplementary file 1 — FIG. S1 Trajectories of individual MDS‐UPDRS Part I total scores (A) and changes in total scores (B) from baseline to the end of the observation period in all patients (n = 996). MDS‐UPDRS, Movement Disorder Society Unified Parkinson's Disease Rating Scale. FIG. S2. Number of NMSs at each time point by the following 3 groups: unchanged (A), deteriorated (B), and improved (C). NMSs, nonmotor symptoms. FIG. S3. Odds ratio with 95% Wald confidence limits (multivariate analysis). LD, levodopa‐containing drug; MDS‐UPDRS, Movement Disorder Society Unified Parkinson's Disease Rating Scale; mH&Y, modified Hoehn and Yahr scale; PD, Parkinson's disease. [file MDC3-7-431-s001.pdf]

Supplementary Figure S1.

A)

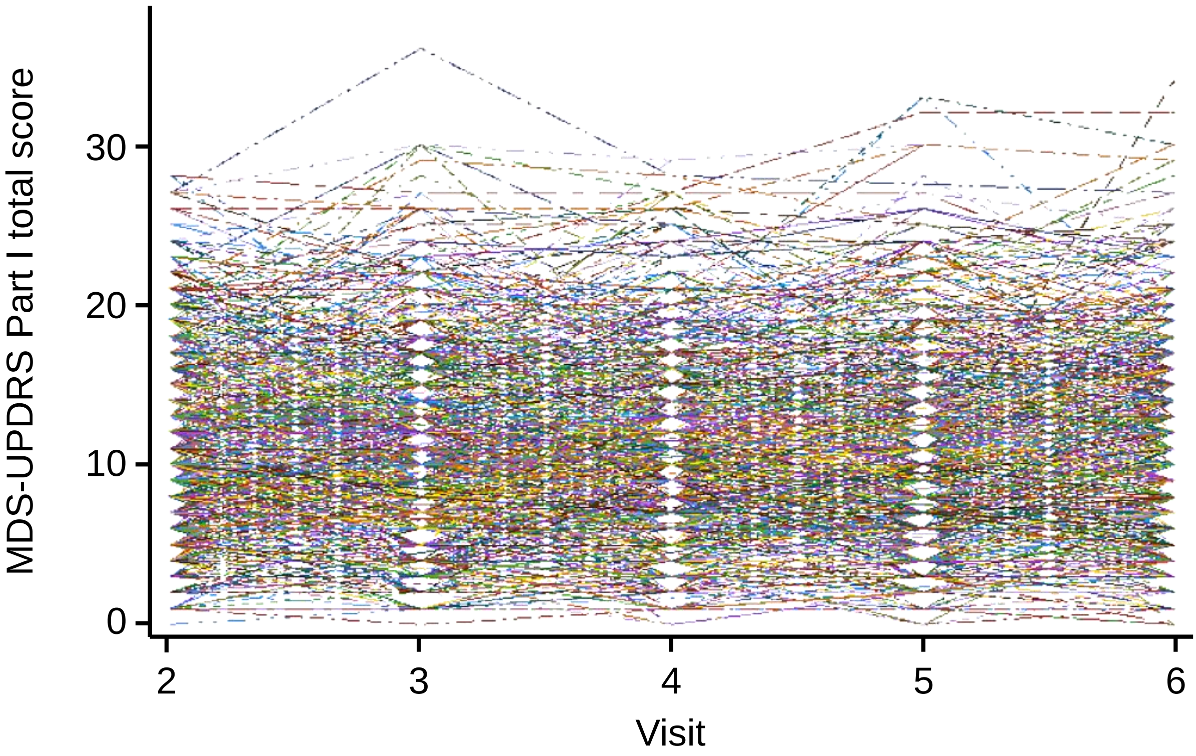

MDS-UPDRS, Movement Disorder Society Unified Parkinson’s Disease Rating Scale

Supplementary Figure S1.

B)

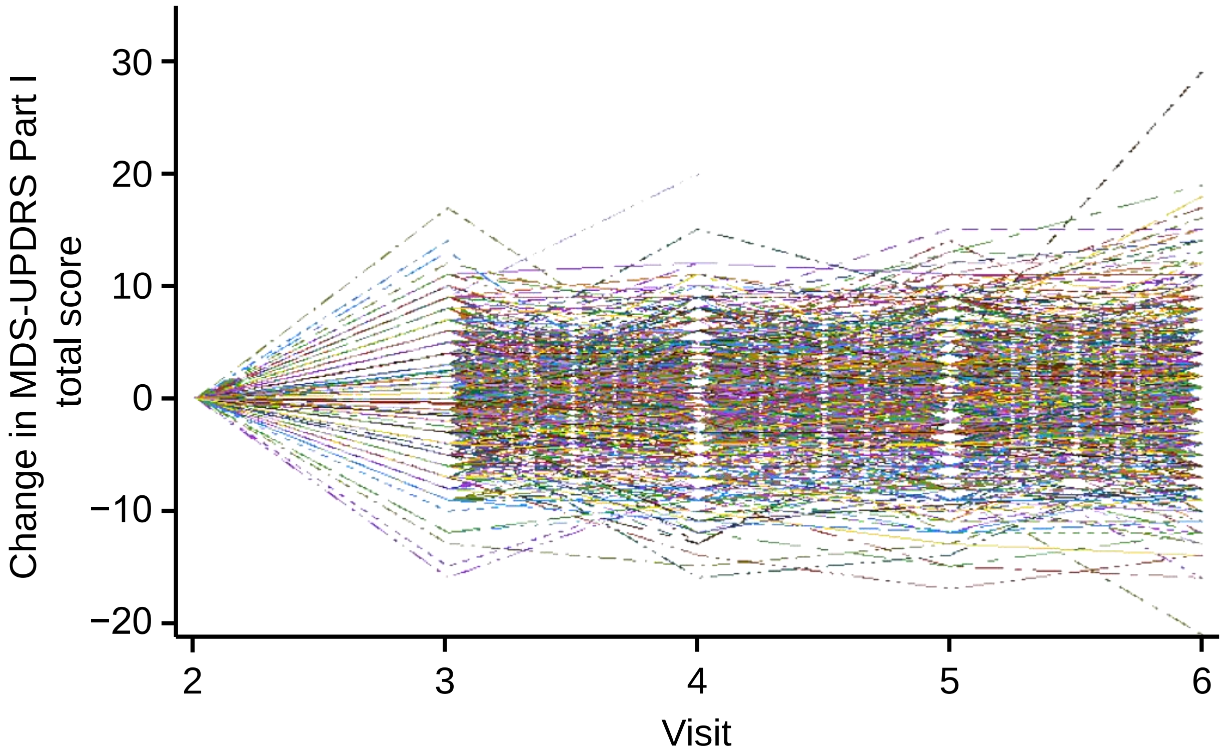

MDS-UPDRS, Movement Disorder Society Unified Parkinson's Disease Rating Scale

Supplementary Figure S2.

A) Unchanged

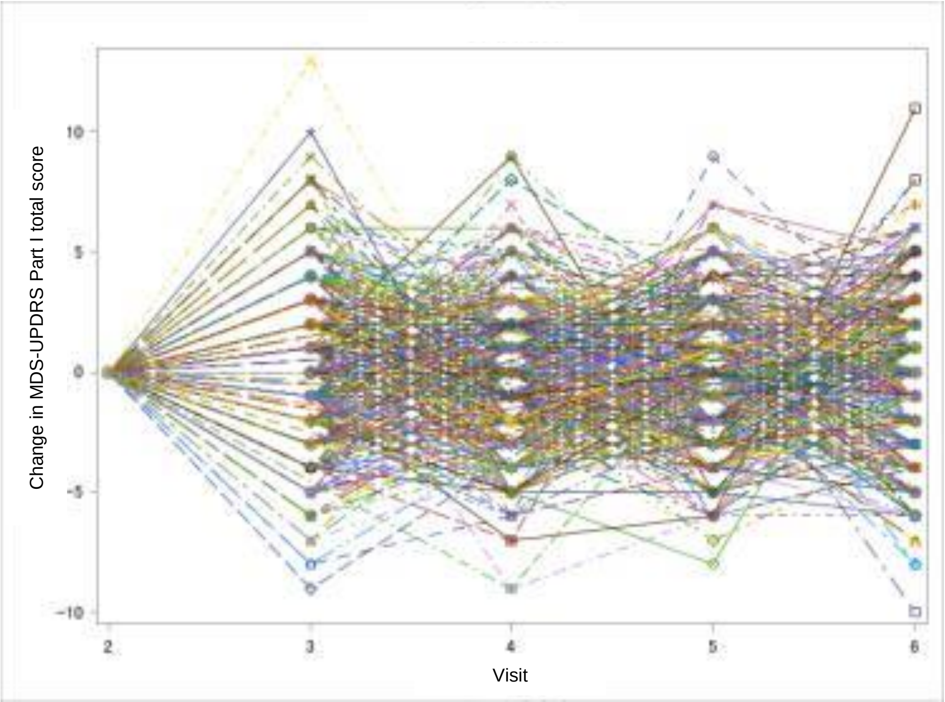

NMS, non-motor symptoms

Supplementary Figure S2.

B) Deteriorated

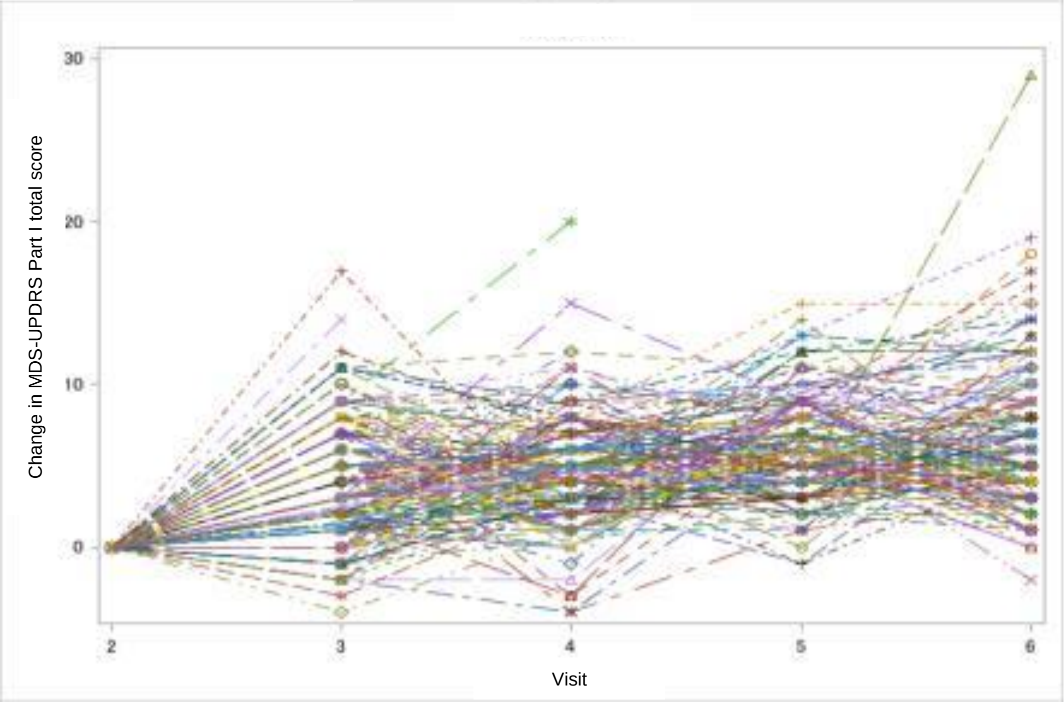

NMS, non-motor symptoms

Supplementary Figure S2.

C) Improved

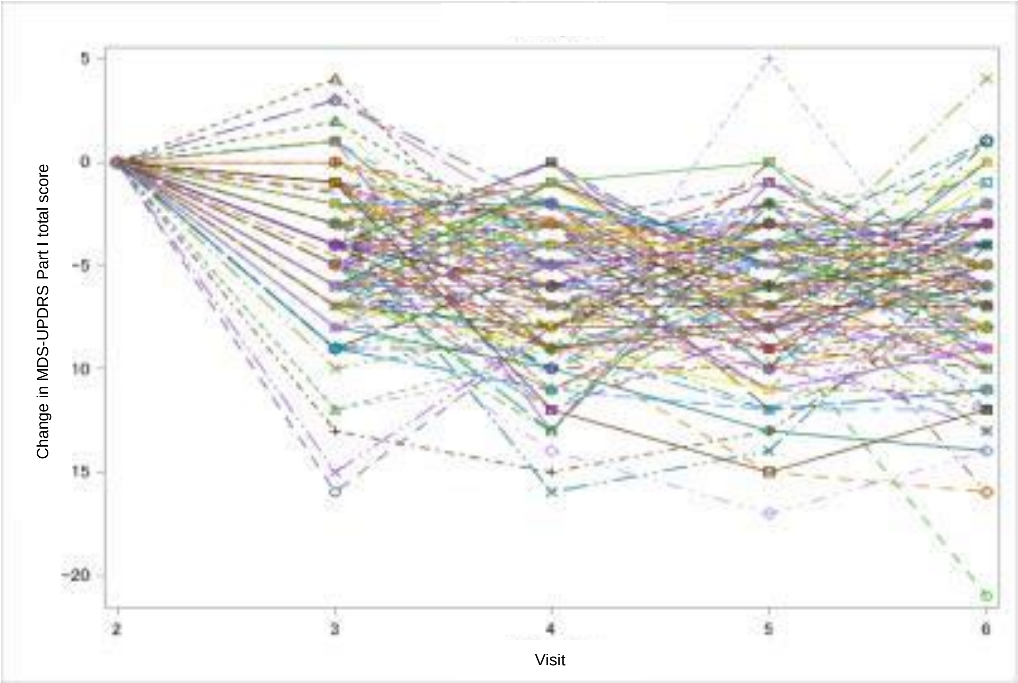

NMS, non-motor symptoms

Supplementary Figure S3.

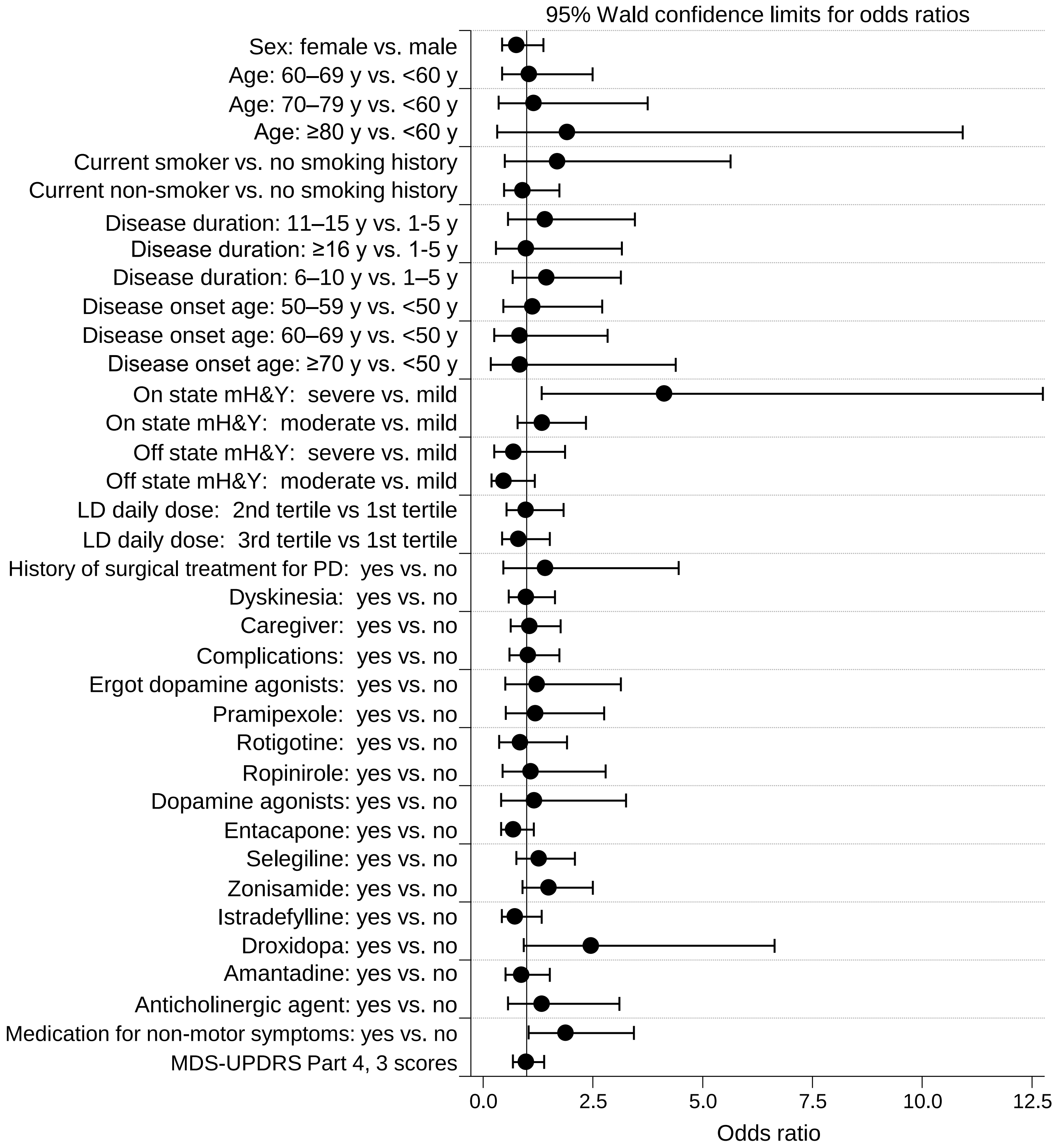

LD, levodopa-containing drug; MDS-UPDRS, Movement Disorder Society Unified Parkinson's Disease Rating Scale; mH&Y, modified Hoehn and Yahr scale; PD, Parkinson's disease
